# *Plasmodium vivax* Seroprevalence in Bred Cynomolgus Monkeys, China^1^

**DOI:** 10.3201/eid1710.110719

**Published:** 2011-10

**Authors:** David B. Elmore

**Affiliations:** DBE Veterinary Consulting, San Diego, California, USA

**Keywords:** cynomolgus monkey, malaria, Plasmodium vivax, seroprevalence, parasites, nonhuman primate, macaque, China, letter

**To the Editor:** Having worked with numerous species of research nonhuman primates over the past 26 years, I have a keen interest in related occupational health and safety. In this regard, I was quite interested in the recent report by Li et al. (*1*) and have some comments and questions relative to this article.

The occurrence of *Plasmodium* spp. infection in feral primates, feral source captive primates, or primates bred outdoors in malaria-endemic areas is not uncommon. However, with the exception of *P*. *knowlesi*, it is my understanding that malarial organisms found in cynomolgus monkeys do not pose a major zoonotic concern (although this can always change). Furthermore, it is my understanding that *P. vivax* does not infect macaques, including cynomolgus monkeys.

Other malarial parasites of cynomolgus monkeys, apart from *P. knowlesi*, may include *P. cynomologi*, *P. inui*, *P. fieldi*, and *P. coatneyi*. A recent publication reported that in wild-source cynomolgus monkeys in Malaysia, >90% of the animals tested were positive for >1 *Plasmodium* species. Furthermore, >80% of samples from these animals were positive by specific PCR for >1 of these organisms (*2*).

Using PCR for *Plasmodium* spp. identification, I have tested newly imported research cynomolgus monkeys from various breeding centers in China. I can confirm that some animals have subclinical malarial infections.

Except for the report by Li et al. (*1*), I am unaware of other reports of *P. vivax* in cynomolgus monkeys. It would be interesting to confirm the presence of this organism by using PCR primers specific for *Plasmodium* spp. My questions to the authors relate to the test method used in their study. Was an ELISA for detecting *P. vivax* antibodies the only diagnostic method used to identify this parasite? It may be useful to re-address the specificity of this test in differentiating various *Plasmodium* spp. Until these issues are clearly addressed, their reported results are not reliable.
